# Global Distribution and Diversity of Prevalent Sewage Water Plasmidomes

**DOI:** 10.1128/msystems.00191-22

**Published:** 2022-09-07

**Authors:** Frederik Teudt, Saria Otani, Frank M. Aarestrup

**Affiliations:** a Research Group for Genomic Epidemiology, Technical University of Denmark, Kgs. Lyngby, Denmark; California State University, Fresno

**Keywords:** sewage water plasmidome, AMR, replication initiator proteins, bacteriophages, metagenomics

## Abstract

Sewage water from around the world contains an abundance of short plasmids, several of which harbor antimicrobial resistance genes (ARGs). The global dynamics of plasmid-derived antimicrobial resistance and functions are only starting to be unveiled. Here, we utilized a previously created data set of 159,332 assumed small plasmids from 24 different global sewage samples. The detailed phylogeny, as well as the interplay between their protein domains, ARGs, and predicted bacterial host genera, were investigated to understand sewage plasmidome dynamics globally. A total of 58,429 circular elements carried genes encoding plasmid-related features, and MASH distance analyses showed a high degree of diversity. A single (yet diverse) cluster of 520 predicted Acinetobacter plasmids was predominant among the European sewage water. Our results suggested a prevalence of plasmid-backbone gene combinations over others. This could be related to selected bacterial genera that act as bacterial hosts. These combinations also mirrored the geographical locations of the sewage samples. Our functional domain network analysis identified three groups of plasmids. However, these backbone domains were not exclusive to any given group, and Acinetobacter was the dominant host genus among the theta-replicating plasmids, which contained a reservoir of the macrolide resistance gene pair msr(E) and mph(E). Macrolide resistance genes were the most common in the sewage plasmidomes and were found in the largest number of unique plasmids. While msr(E) and mph(E) were limited to Acinetobacter, erm(B) was disseminated among a range of Firmicutes plasmids, including Staphylococcus and Streptococcus, highlighting a potential reservoir of antibiotic resistance for these pathogens from around the globe.

**IMPORTANCE** Antimicrobial resistance is a global threat to human health, as it inhibits our ability to treat infectious diseases. This study utilizes sewage water plasmidomes to identify plasmid-derived features and highlights antimicrobial resistance genes, particularly macrolide resistance genes, as abundant in sewage water plasmidomes in Firmicutes and Acinetobacter hosts. The emergence of macrolide resistance in these bacteria suggests that macrolide selective pressure exists in sewage water and that the resident bacteria can readily acquire macrolide resistance via small plasmids.

## INTRODUCTION

The effectiveness of antibiotic treatment is under threat from the ongoing resistance epidemic, making antimicrobial resistance (AMR) a major problem for global health (1).

The AMR epidemic is caused in part by the transmission of antimicrobial resistance genes (ARGs) between different bacterial clones and species (2, 3). Plasmids are one of the most common and easily identifiable types of ARG-carrying mobile genetic elements (4). Plasmids often contain beneficial genes that the host microbe can use to accessorize its genome, and these genes allow the microbe to adapt to various factors, such as the presence of metals or antibiotics, and biosynthetic and carbohydrate functions (5, 6).

Conventional methods to study AMR are based on phenotypic testing (e.g., isolating bacteria from samples and examining their growth in the presence of antibiotics) (7). There are two main challenges of phenotypic experiments. First, the need to isolate and cultivate bacteria introduces a selection bias, as only culturable bacteria can be investigated in an experiment. Second, the amount of antibiotics that are tested will be a limiting factor, as each antibiotic has to be individually tested and evaluated, which adds to the workload and resources required for the experiment. To overcome these challenges, this study employs third-generation sequencing to find the ARGs that are responsible for resistance, circumventing the need for phenotypic testing. This strategy has its drawbacks, too, as AMR can only be detected if the gene has been characterized, and innate resistance cannot be detected, as it is not derived from the acquisition of a specific ARG. However, the focus on horizontally transferrable ARGs is an intentional aspect of this study, and comprehensive databases of ARGs are available to aid in the identification of those genes (8, 9).

Plasmids are known to have a high plasticity that increases the bacterial host fitness, which makes them widely transferable between hosts and results in increased plasmid diversity in bacterial communities. Previous studies have shown an extremely high variability of plasmids within and between reservoirs (10, 11). An example of the former is the plasmid-encoded component of the pangenome of E. coli. There is far greater variation in the plasmid-encoded component than in the chromosome-encoded component of the pangenome. Additionally, the plasmid variation is explained not by pure phylogeny, but by niche-phylogeny interaction (10). A concrete example of how niche shapes plasmid gene content has been shown in the family Lactobacillaceae. Plasmids of bacteria adapted to living in vertebrates contain predominantly anaerobic metabolic genes, while plasmids from free-living bacteria contain aerobic metabolic genes. Thus, the availability of oxygen clearly affects the plasmid gene content (11). It also appears that some plasmids are transmitting ARGs globally, as was seen in the recent emergence of the colistin resistance gene mcr-9. This gene is found on similar IncHI2-ST1 plasmids in Europe, North America, and China, suggesting a common ancestry (12). Thus, studying and understanding the global diversity and the similarity of plasmids from metagenomic samples could provide novel insight into the genomic epidemiology of microbial genes, including ARGs.

Obtaining comparable samples from global sources is, however, a challenge. In recent years, urban sewage has been used as a comparable matrix to successfully study the global occurrence of antimicrobial resistance, the virome, and human populations (13–15). AMR in sewage reflects both the selective pressure in the general environment from which the sewage runs off, and the selective pressure caused by the presence of ARGs in the sewage (e.g., the usage of macrolide antibiotics leads to the presence of macrolide antibiotics in the sewage water itself) (16). We recently applied long read sequencing to explore the occurrence and diversity of the small plasmidome in 24 globally obtained sewage samples and assembled 165,302 contigs (159,322 circular), of which 58,429 carried genes encoding plasmid-related proteins and 11,222 carried genes encoding virus-related or phage-related proteins (17). These plasmidomes were compared to conventional whole community metagenomic extractions from the corresponding samples, elucidating which AMR classes were preferentially found in the plasmidome of sewage communities. One of the highlights of this study was the abundance of macrolide resistance genes in the plasmidomes, including the erm(B), mph(E), mef(A), and msr(D) genes.

Here, we investigate these data further with gene network analysis. Gene networks are often employed to understand biological systems, mainly gene regulatory networks (18, 19). Gene networks have also been used to classify plasmids into taxonomic units and to trace horizontal gene transfer in our data set (20, 21). We also aimed to find gene clusters by looking for conserved regions at the domain level. We employed gene network analysis to find patterns of domain usage in sewage water plasmids. Finally, we applied machine learning tools to link plasmid sequences to their most likely bacterial hosts and to investigate the interplay between AMR, hosts, and backbone proteins.

## RESULTS

### Length distribution of circular assemblies.

**FIG 1:**
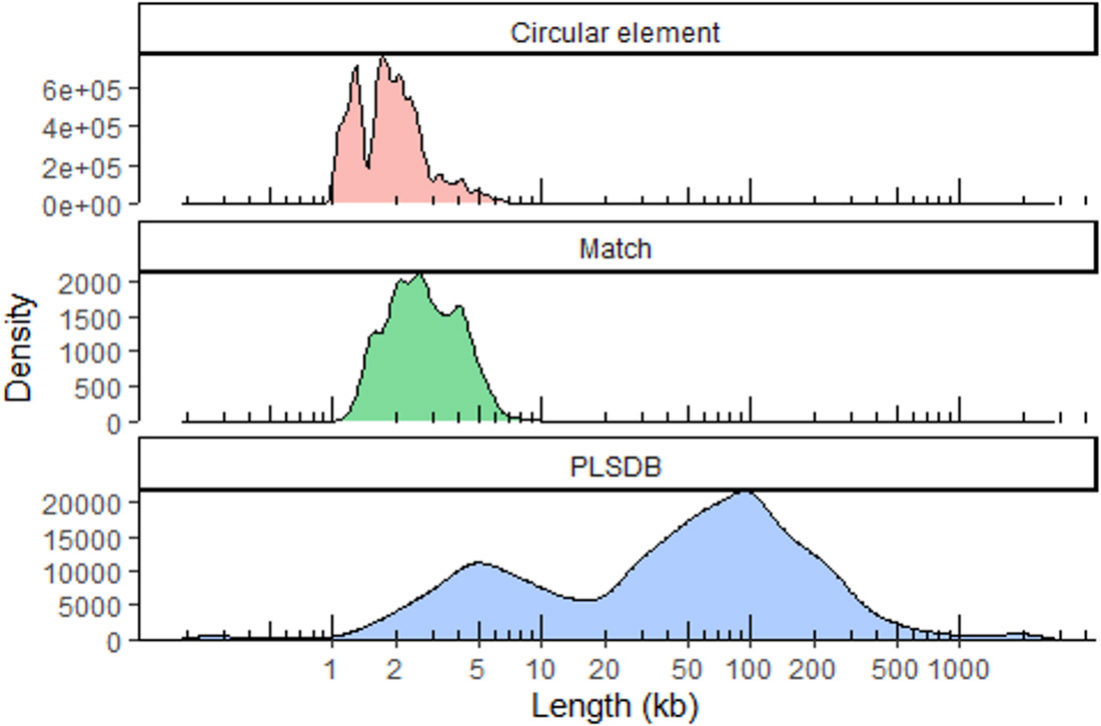
Length distributions of circular elements in our samples before homology reduction (top), PLSDB plasmids that match plasmids found in our samples (middle), and all PLSDB plasmids (bottom).

Sewage water plasmids from 24 samples from 22 countries around the world (Table S1), which have been described previously (17), were compared to the PLSDB plasmid database. The circular elements (Fig. 1, top panel) were predominantly smaller than the PLSDB plasmids.

10.1128/msystems.00191-22.2 TABLE S1 List of the sewage samples that are included in the study. The data are available under ENA project accession number PRJEB41171 (finished assemblies are ERZ1694234 through ERZ1694257). Download Table S1, XLSX file, 0.01 MB. Copyright © 2022 Teudt et al. 2022 Teudt et al. https://creativecommons.org/licenses/by/4.0/ This content is distributed under the terms of the Creative Commons Attribution 4.0 International license.

The plasmids from PLSDB (22) were generally quite large (Fig. 1, bottom panel). The PLSDB plasmids were split into two main types: small plasmids of lengths of around 5 kb, and large plasmids ranging from 20 to 300 kb. The size range of the PLSDB plasmids that matched circular elements in our samples were in the size range of approximately 1,500 bp to 5,000 bp (Fig. 1, middle panel).

Overall, we are looking at a segment of plasmids that might currently be under-researched.

### Phylogenetic analyses.

**FIG 2:**
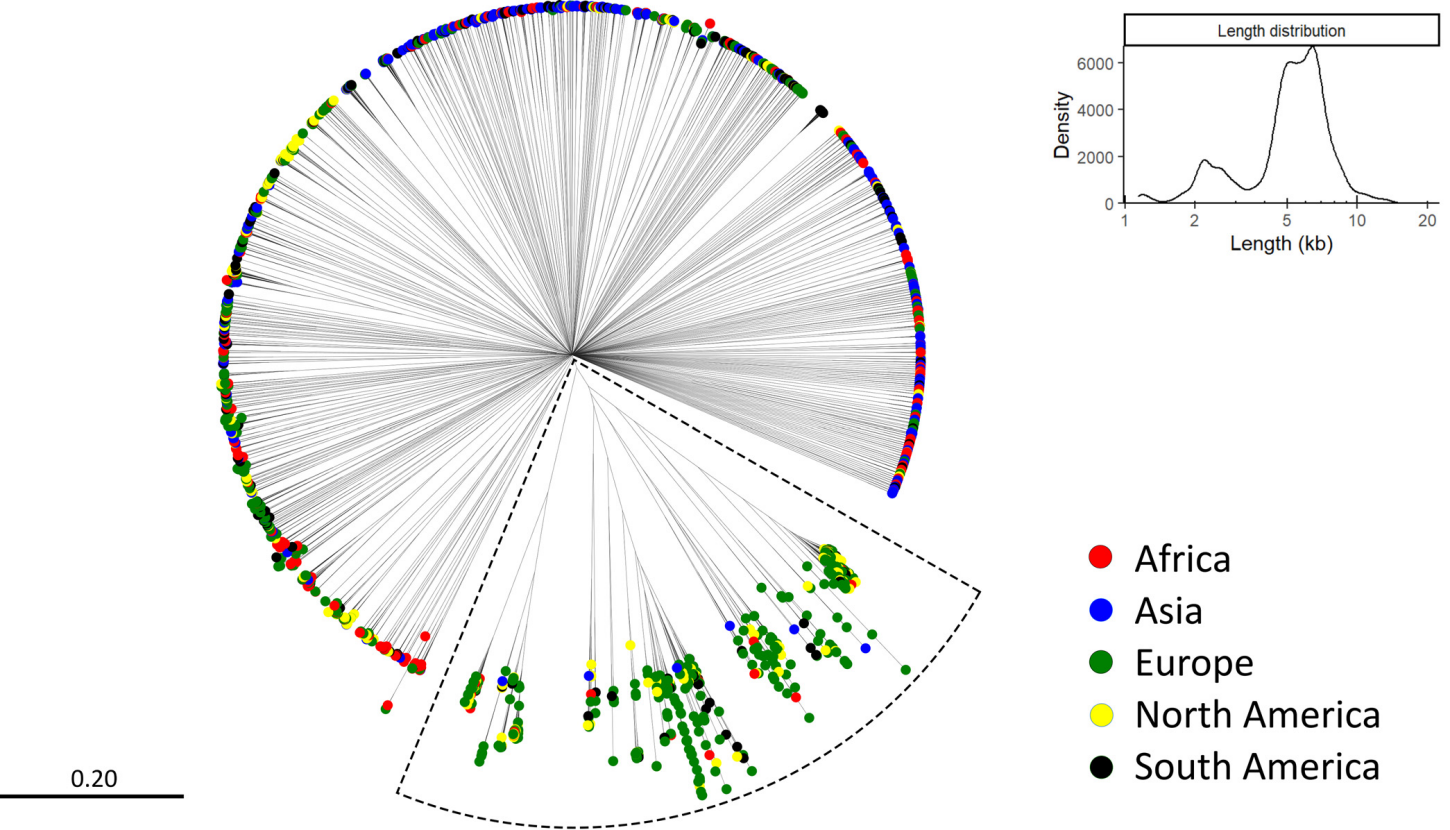
Dendrogram of the longest 100 circular elements from each sample. 140 singletons were removed, leaving 2,060 plasmids. Elements only connected through the center had no similarity. The length distribution of the circular elements is shown in the top right insert. The continent of origin of each plasmid is represented by its color.

After individual homology reduction of the 24 samples, the longest 100 circular elements from each sample were compared in phylogenetic analyses via a neighbor-joining tree algorithm (Fig. 2). With the exception of a smaller subset of predominantly European samples, indicated by dotted lines, there was little similarity between the analyzed circular elements (Fig. 2), despite the similar sizes of the tested circular elements.

The 24 samples were distributed across the continents as follows: four from Africa, five from Asia, eight from Europe, three from North America, and four from South America. For this reason, the overrepresentation of European plasmids is expected. However, the cluster in the dotted box in Fig. 2 contains a proportion of European plasmids that exceeds one-third and matches the proportion of samples that are from Europe. However, there are also African, Asian, North American, and South American plasmids in the cluster, and this cluster was chosen for further downstream analyses.

### Acinetobacter plasmids from sewage water.

**FIG 3:**
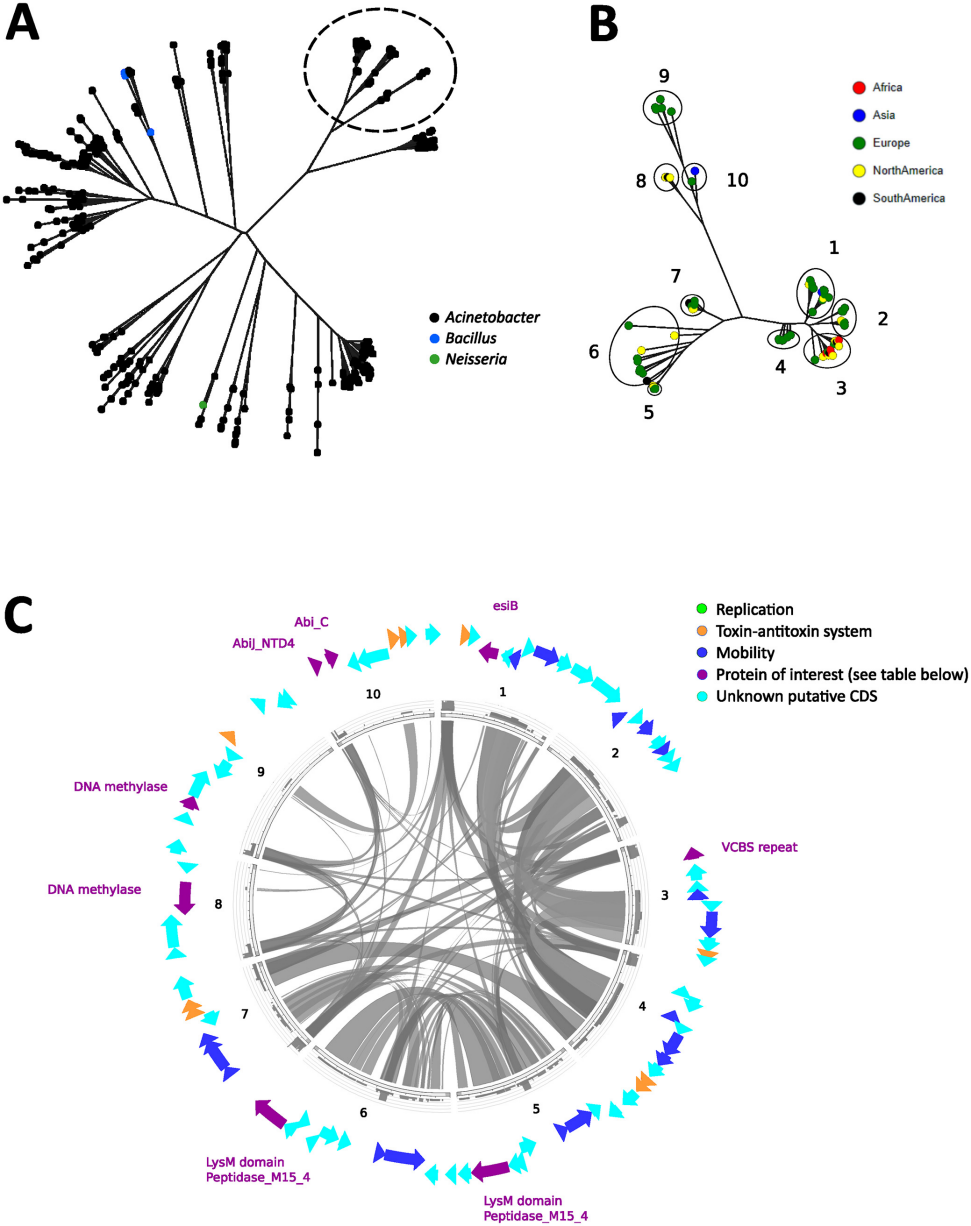
Visualizations of putative Acinetobacter plasmids. (A) Dendrogram of the European cluster, colored by genus. A subtree that was selected for closer inspection is circled in black. (B) Dendrogram showing the geographical distribution of the selected Acinetobacter plasmids. The plasmids were divided into numbered groups from 1 to 10 for alignment-based comparison. (C) Circos plots of representatives from each of the 10 groups. Note that no replication genes were found. The functions of the genes highlighted in purple text are given in Table 1.

The cluster of interest chosen from Fig. 2 was dominated by plasmids predicted to be Acinetobacter, with only a few *Neisseria* or *Bacillus* plasmids (Fig. 3A). The plasmids in the cluster numbered too many to allow for the visualization of their genes and the alignments between them, so an even smaller subset was selected for visualization. This subset was composed exclusively of plasmids predicted to be Acinetobacter (Fig. 3A, dotted circle). A majority of the plasmids were European, but all of the continents were still represented (Fig. 3B). A plasmid from each branch of the subset was annotated and aligned against the other plasmids (Fig. 3C).

This cluster is of interest because it contains no known replication mechanisms. This is despite the fact that it contains other predominantly known plasmid-related functions, such as mobility systems and toxin-antitoxin systems (23, 24). The lack of replication proteins in these genetic elements indicates that this group of circular elements are not plasmids, are plasmids without replication initiator proteins, or are plasmids with new types of replication initiator proteins.

Fig. 3C shows the predicted ORFs in representatives from the subset, and Table 1 lists proteins of interest. Many of the ORFs either did not have a predicted Pfam associated or had Pfams with uncertain function, such as various helix-turn-helix domains. The proteins with assignable functions involved phage protection systems (N6_N4_Mtase [25], N6_Mtase [26–28], AbiJ_NTD4 [29], and Abi_C [30]), cell wall metabolism (LysM [31, 32] and Peptidase_M15_4 [33, 34]), immune escape (esiB [35]), and cell-cell signaling (VCBS [36]).

**TABLE 1:** Protein families predicted on the Acinetobacter cluster (purple CDS in Fig. 3C)

Gene or domain	Long name	Function
EsiB	Secretory immunoglobulin A-binding protein	Inhibition of immunoglobulin A
PF13517 VCBS (FG-GAP_3)	Repeat domain in *Vibrio*, *Colwellia*, *Bradyrhizobium* and *Shewanella* (Also known as FG-GAP-like repeat)	Diverse range of cell-cell interactions
PF01476 LysM PF13539 Peptidase_M15_4	Lysin Motif d-alanyl-d-alanine carboxypeptidase	Peptidoglycan binding Cell wall metabolism
PF01555 N6_N4_Mtase	DNA methylase	Host DNA protection against restriction enzymes
PF02384 N6_Mtase	N−6 DNA Methylase	Host DNA protection against restriction enzymes
PF18863 AbiJ_NTD4	AbiJ N-terminal domain 4	Bacteriophage immunity
PF14355 Abi_C	Abortive infection C terminus	Bacteriophage immunity

### Clustering of circular elements into plasmids and bacteriophages.

**FIG 4:**
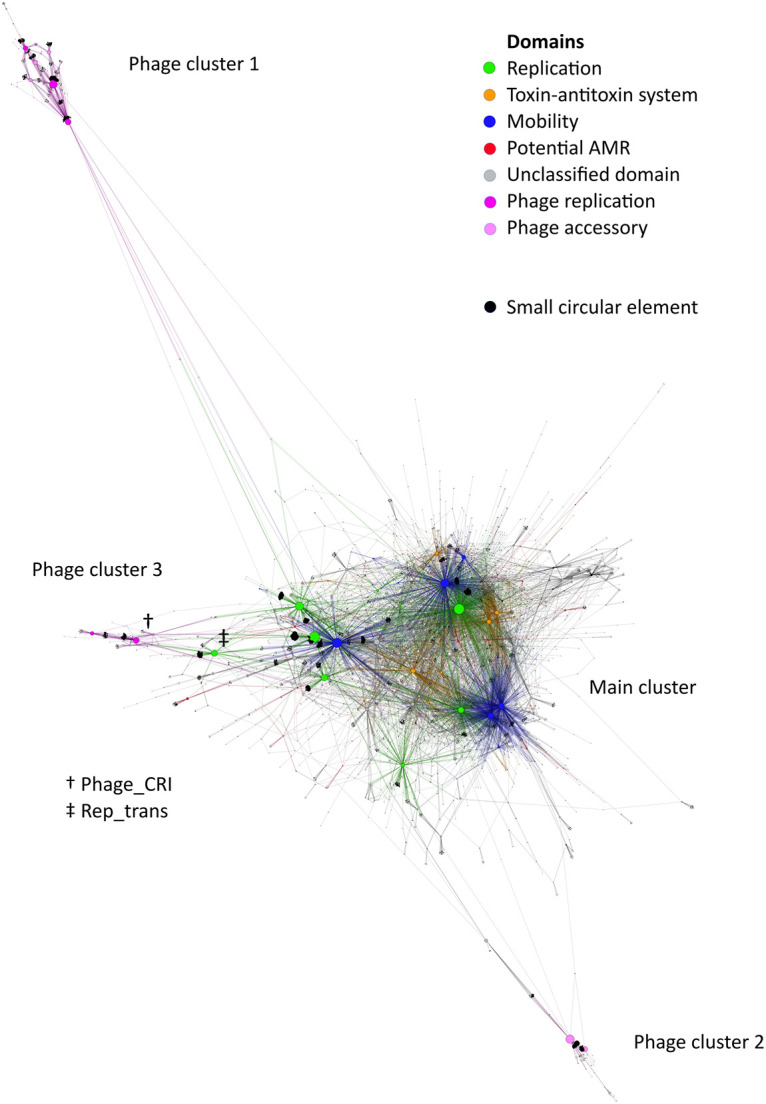
Bipartite network graph of sewage water Pfam domains. Layout by ForceAtlas2, with node sizes ordered by count. The sequences separate into four distinct clusters: one main plasmid cluster and a viral cluster at the top (phage cluster 1), at the bottom (phage cluster 2), and to the left (phage cluster 3, centered around the Phage_CRI domain). Phage cluster 3 was not well-separated from the plasmids due to its stronger association with the replication initiator domain of the main cluster, most noticeably Rep_trans.

To investigate the circular elements at large, we made a network graph of all domains to trace their interactions in the global environment. The network layout was calculated by the ForceAtlas2 (37) algorithm. The layout revealed one main plasmid cluster and two clearly distinct bacteriophage clusters. The main cluster contained most of the nodes, while phage clusters 1 and 2 were in the periphery (Fig. 4). Interestingly, the two clusters were both apparently ssDNA viruses, as indicated by their domains (38–40). One cluster contained viruses with the Gemini_AL1 and Viral_Rep replication domains, which may primarily infect eukaryotes (39) (Fig. 4, phage cluster 1), whereas the other cluster contained no evident replication proteins but did contain the scaffolding protein Chlamy_scaf (38) (Fig. 4, phage cluster 2) and the capsid domain Phage_F (40) (pink), both of which are found in ssDNA bacteriophages.

There is one last bacteriophage cluster, but it is less clearly separated from the main cluster (Fig. 4, phage cluster 3). The majority of the viral accessory proteins of this cluster are either associated with the Phage_CRI or, to a lesser extent, the Phage_X viral replication domains found in this cluster. However, some are associated with the replication initiation domains of the main plasmid cluster, explaining why phage cluster 3 fails to separate from the main cluster. This is most noticeable from the fact that the Rep_trans domain has been pulled halfway from the main cluster toward phage cluster 3.

### Comparison of plasmid replication groups.

Plasmid replication initiators were predictably the most common domains in the data set (Fig. 4, green nodes) with mobilization domains as a close second (Fig. 4, blue nodes). Replication and mobilization genes separate into three clusters in the network, with 2 to 4 major domains in each. Group 1 is composed of Rep_1, Rep_2, RepL, and Mob_Pre, and it is the most prevalent. Group 2 is composed of Rep_3 and MobA_MobL. Group 3 is composed of Replicase, Relaxase, and MobC, and it is the least prevalent. There were more backbone proteins, but these were selected due to two factors. First, they needed to be large enough to be identifiable in the network as a major backbone domain. Second, they needed to be unambiguously clustered with other backbones in their vicinity. Rep_trans is closest to Group 1, but it is not included in the group. Of the 72,225 circular elements, 34,659 contained Pfam domains, and 21,639 had plasmid backbone proteins from at least one of the three replication groups.

**FIG 5:**
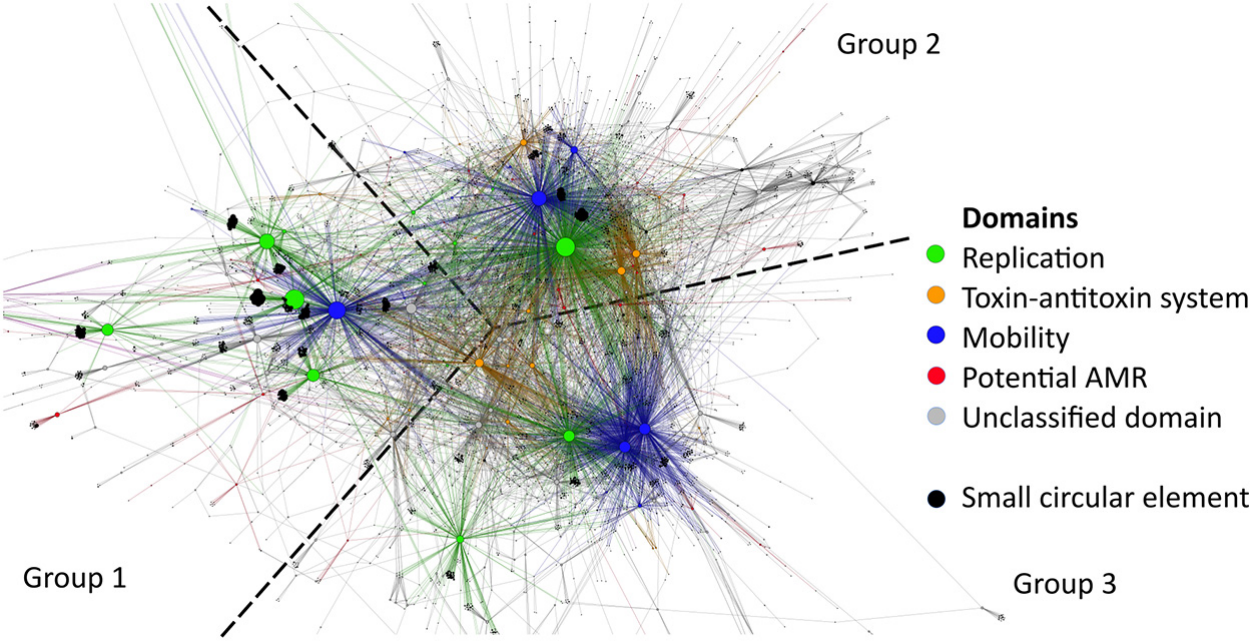
Plasmid region of the bipartite network graph of sewage water Pfam domains. Layout by ForceAtlas2, with node sizes ordered by count. The backbone proteins (replication and mobilization, green and blue, respectively) can be separated into three groups.

Replication group 1 contained domains for rolling circle initiator proteins (Rep_1 [41], Rep_2 [42, 43], RepL [44, 45], and the excluded Rep_trans [46]), while groups 2 and 3 contained domains of theta replication initiators (Rep_3 [47, 48] and Replicase [49]). Therefore, it makes sense that the group 1 replication domains are the ones that are the most entangled with bacteriophages, which also use rolling circle replication (50). The rolling circle-replicating section of the network generally consisted of a few large nodes, while the theta-replicating section was more dispersed (Fig. 5). Most of the major backbone domains had a large cluster of circular elements connected to them: the elements with only a single backbone domain found on them. These were the most common types of circular elements.

Closer inspection of the backbone genes showed that there is minor overlap between the backbone clusters (Table 2). As expected, not all replication proteins were associated with a mobilization protein. However, if replication proteins were associated with mobilization proteins, they would predominantly belong to specific Pfams. Two different replication proteins were almost never found on the same plasmid (Table 2). Likewise, different mobilization domains were generally not found together on the same plasmid, with the exceptions of Relaxase and MobC, which serve complementary functions during plasmid mobilization (Table 2).

**TABLE 2:**
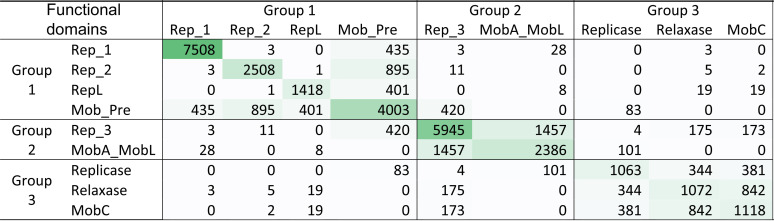
Backbone types of plasmids. Numbers indicate the amount of times two domains were observed on the same plasmid^*a*^

a Rep_1, Rep_2, RepL, Rep_3, and Replicase are replication initiator domains. Mob_Pre, MobA_MobL, Relaxase, and MobC are mobilization-related domains.

The division of backbone genes into groups was based on the observed trend in the network, but it was not a perfect division of the domains. Replication proteins of group 2 and group 3 most often mixed with mobilization domains of other groups, while group 1 replication proteins predominantly associated with Mob_Pre.

Besides replication initiator proteins and mobilization proteins, the network also showed one other type of common domain: toxin-antitoxin systems. These were more closely associated with the theta-replicating plasmids (Fig. 5, orange nodes). Toxin-antitoxin systems were not as common as replication and mobility domains, but they were far more common than any other type of domain.

### ARGs and backbones.

After grouping the backbone proteins into three categories, the ARGs associated with each type were investigated. Table 3 shows the backbones as they occur on plasmids that carried ARGs. Interestingly, many ARGs were found on group 1 plasmids, but not those carrying Rep_1, despite it being the most prevalent replication protein. The most prevalent backbone domain on ARG-carrying plasmids was Mob_Pre (of the group 1 backbones). The next most prevalent protein was Rep_3 (of the group 2 backbones). Overall, it was clear that ARGs associated with specific backbone proteins rather than all of the proteins in a backbone group.

**TABLE 3:**
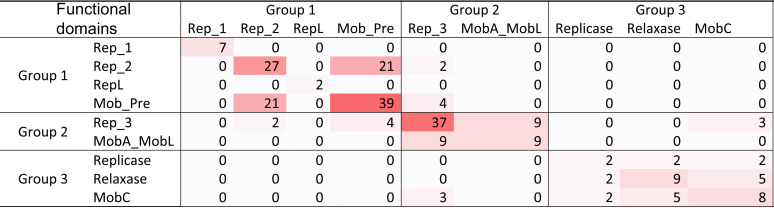
Backbone types of ARG-carrying plasmids^*a*^

a The numbers indicate the amount of times two domains were observed on the same AMR-plasmid.

Bacteriophages usually do not carry ARGs. To discover if this was the case in our samples, we investigated whether there were any ResFinder hits found on the same circular elements that carried bacteriophage proteins. No bacteriophage domains were found alongside ResFinder hits.

Fig. S3 and S4 show the locations of the most commonly found ARG domains in the domain network with respect to the predicted host genus and the sample origin. The domains RrnaAD, Aminoglyc_resit, and CAT were more closely associated with the rolling circle plasmids, while APH MFS_1, and ABC_tran were found with the theta-replicating plasmids. Pentapeptide_4 was located on the outskirts of the plasmid clusters. Table 4 lists the commonly found ARGs to which these domains correspond.

**TABLE 4:** Geographical distribution of the most common ARGs along with the names of their most significant Pfam domains

ARG	Africa	Asia	Europe	North America	South America	Total	Pfam
erm(B)	2	2	20	5	6	35	RrnaAD
msr(E)	3	0	17	0	0	20	ABC_tran
mph(E)	2	0	17	0	0	19	APH
qnrB19	1	1	1	3	8	14	Pentapeptide_4
erm(T)	4	0	3	1	0	8	RrnaAD
lnu(A)	0	2	3	0	1	6	Aminoglyc_resit
tet(39)	1	0	2	0	2	5	MFS_1
aph(3′)-III	0	0	0	0	3	3	APH
cat	0	0	3	0	0	3	CAT

10.1128/msystems.00191-22.9 FIG S3 The location of the most common ARG domains in the domain network with respect to the predicted host genus. Download FIG S3, TIF file, 0.9 MB. Copyright © 2022 Teudt et al. 2022 Teudt et al. https://creativecommons.org/licenses/by/4.0/ This content is distributed under the terms of the Creative Commons Attribution 4.0 International license.

10.1128/msystems.00191-22.10 FIG S4 The location of the most common ARG domains in the domain network with respect to the sample origin. Download FIG S4, TIF file, 0.9 MB. Copyright © 2022 Teudt et al. 2022 Teudt et al. https://creativecommons.org/licenses/by/4.0/ This content is distributed under the terms of the Creative Commons Attribution 4.0 International license.

### AMR and bacterial hosts.

The hosts of the ARG-carrying plasmids were also predicted in the global sewage plasmidomes (Table S3). We had a total of 150 ARGs found after homology reduction. Using a plasmid host species predictor (51), we investigated the host range of the ARG-carrying plasmids. The ARG-carrying plasmids were limited to 14 different predicted genera.

10.1128/msystems.00191-22.4 TABLE S3 Plasmid-encoded AMR genes and their predicted host organisms. AMR-containing plasmids had the genera of their hosts predicted. Counts indicate the amount of times a plasmid-encoded AMR gene was found in a given genus. Download Table S3, XLSX file, 0.01 MB. Copyright © 2022 Teudt et al. 2022 Teudt et al. https://creativecommons.org/licenses/by/4.0/ This content is distributed under the terms of the Creative Commons Attribution 4.0 International license.

Interestingly, the most common ARGs in the homology-reduced plasmids were capable of conferring macrolide resistance, but in different host ranges (Table S3). The most commonly found ARG was erm(B). erm(B) is a methyltransferase that confers macrolide resistance by methylating rRNA (52). It was found in five different genera of Firmicutes. The next most common genes were the gene pair msr(E) and mph(E). msr(E) is a ribosome protection protein that confers resistance to a wide range of ribosome-targeting antibiotics (e.g., macrolides, lincosamides, and phenicols) (53). mph(E) is a macrolide phosphotransferase that confers resistance to macrolides (54). The other AMR genes found on more than two plasmids were qnrB19 in Klebsiella and Salmonella (which confers resistance to quinolones) (55), erm(T) in Staphylococcus, *Lactiplantibacillus*, and Streptococcus, lnu(A) in Staphylococcus and *Lactiplantibacillus*, tet(39) in Acinetobacter, and cat and aph(3′)-III in Escherichia.

### Genera and backbones.

Having found a link between ARGs and host bacterial genera, we then investigated the relationship between the bacterial host genus and the plasmid backbone type. The host predictions are listed in Table S2. The five most often predicted bacterial host genera were analyzed for their backbone gene content. Importantly, hosts were predicted for all circular elements, including bacteriophages. Two different methods for excluding phages were tried, but they were both found to be inaccurate (Fig. S1 and S2). However, bacteriophages should not have plasmid backbone genes, so their output should not skew the backbone composition analysis.

10.1128/msystems.00191-22.3 TABLE S2 List of plasmid hosts predicted on sewage circular elements. Download Table S2, XLSX file, 0.01 MB. Copyright © 2022 Teudt et al. 2022 Teudt et al. https://creativecommons.org/licenses/by/4.0/ This content is distributed under the terms of the Creative Commons Attribution 4.0 International license.

10.1128/msystems.00191-22.7 FIG S1 PPR-Meta predictions of the DNA sources for sequences with known phage domains (left panel), known plasmid domains (middle panel), and no signature domain (right panel). Download FIG S1, TIF file, 0.01 MB. Copyright © 2022 Teudt et al. 2022 Teudt et al. https://creativecommons.org/licenses/by/4.0/ This content is distributed under the terms of the Creative Commons Attribution 4.0 International license.

10.1128/msystems.00191-22.8 FIG S2 SourceFinder predictions of the DNA sources for sequences with known phage domains (left panel), known plasmid domains (middle panel), and no signature domain (right panel). Download FIG S2, TIF file, 0.01 MB. Copyright © 2022 Teudt et al. 2022 Teudt et al. https://creativecommons.org/licenses/by/4.0/ This content is distributed under the terms of the Creative Commons Attribution 4.0 International license.

*Planococcus* was by far the most often predicted host (*n* = 26,165), but relatively few backbone genes were found on these plasmids (Table S4). Rep_2 and Rep_L made up an unusually large proportion of the backbone genes of these plasmids. Because *Planococcus* is predominantly a marine halophilic genus (56, 57), and because relatively few of these circular elements carried plasmid backbone genes, it is likely that most of these predictions were false, likely stemming from phage contamination.

10.1128/msystems.00191-22.5 TABLE S4 Backbone domains of five bacterial taxa: *Planococcus* (*n* = 26,165), *Neisseria* (*n* = 11,710), *Lactiplantibacillus* (*n* = 10,090), Acinetobacter (*n* = 8,632), and Escherichia (*n* = 5,210). Download Table S4, XLSX file, 0.01 MB. Copyright © 2022 Teudt et al. 2022 Teudt et al. https://creativecommons.org/licenses/by/4.0/ This content is distributed under the terms of the Creative Commons Attribution 4.0 International license.

*Neisseria* was also predicted in high abundance (*n* = 11,710) (Table S5). The abundances of the backbone genes were fairly similar to the abundances in general, though groups 2 and 3 were again slightly underrepresented. *Neisseria* is a common human and animal commensal (58). Thus, it is not unexpected to find it in sewage.

10.1128/msystems.00191-22.6 TABLE S5 Host prediction of erm(B)-carrying plasmids. BLAST and the nonfragment model predicted all of the plasmid hosts to be Firmicutes (with some disagreement about which genera, specifically), while the fragment model predicted a far broader range of hosts, including non-Firmicutes. This is followed by the host prediction of msr(E)-carrying plasmids. BLAST and the nonfragment model predicted the plasmid hosts to be almost exclusively Acinetobacter. Download Table S5, XLSX file, 0.01 MB. Copyright © 2022 Teudt et al. 2022 Teudt et al. https://creativecommons.org/licenses/by/4.0/ This content is distributed under the terms of the Creative Commons Attribution 4.0 International license.

The *Lactiplantibacillus* plasmids were similar to the *Neisseria* plasmids but notably contained many backbone gene combinations across groupings (e.g., Replicase and Mob_Pre) (Table S6). *Lactiplantibacillus* is found as a commensal (59) and is used for silage production (60). Thus, it is likely to be found in sewage water.

Acinetobacter plasmids had a high abundance of group 2 and group 3 backbones. In fact, a majority of the group 3 backbones were predicted to be from Acinetobacter (compare Table 2 and Table S7). Acinetobacter is fairly ubiquitous and is expected in sewage water (61).

Escherichia plasmids had fewer Rep_1 genes than did the population in general, but they were otherwise fairly representative of the population (Table S8). Escherichia is a common gut bacterium (62). Thus, it is likely to be present in sewage, lending credence to its prediction as a host. By far, E. coli had the most varied range of backbone domains.

There was a clear link between genus and plasmid backbone genes, but no genus was predicted exclusively to one group. Coloring the domain network according to predicted host genera also highlighted the association between backbone genes and host genera. Importantly, the theta-replicating plasmids were predominantly Acinetobacter plasmids, whereas the rolling circle replicating plasmids were more varied in their host predictions (Fig. S3). As anticipated, the sequences predicted to be from *Planococcus* were mainly viruses, not plasmids (Fig. S3, purple nodes).

### Geographical distribution of domains and ARGs.

Coloring the domain network according to the continent of origin showed that the theta-replicating plasmid cluster was dominated by European samples, whereas sequences in phage clusters 1 and 2 were predominantly from Asian samples (Fig. S4). The rolling circle plasmids were varied regarding their continents of origin.

The European samples contained a disproportionate amount of the most common ARGs found in the data (Table 4). erm(B) and qnr19 were the only genes detected on all continents. The gene pair msr(E) and mph(E) were found on many plasmids from Europe and on a few plasmids from Africa, exclusively.

The three resistance genes most often encountered in the homology-reduced data set all conferred macrolide resistance. erm(B) was found in Firmicutes, and mph(E) and msr(E) were found in Acinetobacter. Additionally, erm(B) was found across five different genera (Table S3). erm(B) was found on all continents. The samples from Europe contained the most erm(B) genes, followed by those from the Americas, while the samples from Africa and Asia contained the least. However, the African samples contained the most erm(T) genes. mph(E) and msr(E) were predominantly found in Europe (Table 4).

The domains pentapeptide_4 (containing the gene qnrB19) and CAT (containing the gene cat) were not located within replication groups. qnr19 was found in Salmonella plasmids (Table S3); Salmonella plasmids were rarely predicted in the data, but almost all of the qnr19 genes were located on them. qnr had a worldwide distribution (Table 4). cat was found in the European samples (Table 4) in Escherichia (Table S3). Plasmids predicted to be Escherichia were not rare in the data, but they were not associated with either replication group. Like CAT, Escherichia lies in between the groups in the domain network (Fig. S4).

## DISCUSSION

### Assembly properties.

In this study, we further analyzed previously assembled short circular elements, mainly plasmids, from sewage water from around the world (17). 48% of the circular elements encoded functional domains (Pfam). The low proportion was likely due to the low plasmid/chromosome DNA ratio, as potential low copy numbers can make it difficult to detect plasmids, or to the low quality of the Oxford Nanopore reads from which the plasmids were assembled. Of the Pfam-containing elements, 62% contained plasmid replication initiator proteins or mobilization systems, suggesting that our findings are not artifacts.

The lack of longer sequences above 3,000 bp (Fig. 1, top panel) could be caused by a disadvantage of assembling each plasmid from a single read. In this study, read numbers tended to drop with read length. So, assembly is naturally limited at higher ranges, as single reads are less likely to completely span longer plasmids. The smaller peaks in the distribution could be caused by the existence of a few high-abundance plasmids or phages. There were also minor peaks in the distribution of the PLSDB plasmids found in our samples (Fig. 1, middle panel) due to redundancy in PLSDB.

Another likely reason that we observed several small elements (compared to PLSDB) is the presence of other nonplasmid circular elements, notably bacteriophages. This could also be due to a database bias caused by a lack of research into plasmids that are too small to carry genes of interest (e.g., AMR genes).

Previous studies using rolling circle amplification and linear DNA-degradation to isolate plasmids also primarily isolated short sequences (6, 63, 64). These studies employed short read sequencing, in which plasmid assemblies were biased toward shorter ones due to the technology. The use of long read sequencing was used to improve the recovery of larger plasmids. However, it has been shown previously that linear DNA-degradation and rolling circle amplification may introduce a bias toward shorter plasmids (<10 kb) (65).

### Sewage Acinetobacter plasmids.

Acinetobacter was an important sewage water plasmid host in our findings. It has also been described in previous studies as a common sewage bacterium (14). The only major cluster found in our phylogenetic analysis was mainly composed of Acinetobacter plasmids (Fig. 3A), and Acinetobacter was the dominant genus found among replication groups 2 and 3 in the functional analyses (Table S7; Fig. S3). The Acinetobacter plasmids also contained all of the msr(E) and mph(E) macrolide resistance genes that were found.

Of the subset of Acinetobacter plasmids that were chosen for closer analysis, a majority of them were from the European samples (Fig. 3B). With regard to functional domains, replication domains were the most dominant in all plasmids, but they were absent in this plasmid subset (Fig. 3C). It could be that the assemblies are integrative conjugative elements (ICEs). These elements can exist as circular ones and contain genes for conjugation, similar to plasmids; however, they do not contain replication genes (66). Instead, they rely on integration into the host genome for replication. At odds with this theory is the lack of any predicted integrases and excisionases between the sequences. This could also be a case of missed replication genes, as there are regions of homology with no predicted open reading frames (ORFs) (Fig. 3C). Shifting the start point of the sequence does not reveal any ORF in these areas. It should be noted that only a small amount of these sequences are homologous. Thus, it is possible that the lack of replication genes in this cluster is simply a coincidence. Lastly, theta-replicating plasmids, which Acinetobacter plasmids often are (Table S7), can initiate replication with only host factors (67), which could also explain the missing replication genes.

It made sense that we found phage protection functions on our Acinetobacter plasmids, as plasmid-encoded phage defense systems are a known strategy for combating phage infection (68, 69). We also found cell wall metabolism functions. Cell walls are common antibiotic targets, and the genes involved with cell wall metabolism are potential AMR genes. Specifically, the Peptidase_M15_4 found on our subset (Fig. 3C; Table 1) is similar to VanY, a vancomycin resistance gene (33, 34). However, VanY exists in a much larger gene cluster than could possibly fit on these plasmids. So, if these genes somehow confer resistance to a cell wall-targeting antibiotic type, it may not be vancomycin, and it is not a homologous mechanism to VanY.

### Functional domains in sewage plasmidomes.

While the findings from the inspected Acinetobacter subset were interesting, these plasmids represented only a small fraction of the investigated circular elements. The domain network allowed us to investigate the plasmid population at large.

The analysis of the sewage plasmidomes at large was done based on functional Pfam predictions. This methodology intrinsically filtered out small possible artifacts from the assemblies if they contained no Pfams. It also acted as a control for separating plasmids from phages using domain classification.

The Pfam network analysis showed that there were three predominant plasmid replication groups (Fig. 5; Table 2). In each group, there was an association between the replication domain and the mobilization domain. This could be because the replication and mobilization proteins are dependent on the host machinery. Thus, certain domains are favored by certain hosts, and they are therefore found together. The bacterial host genus predictions supported the claim that different bacterial genera favor different backbone domains (Fig. S3). It did not appear to correlate with closely related bacterial taxa, as the *Planococcus* plasmids (of phylum Firmicutes) (Table S4) had similar backbones to those of the *Neisseria* plasmids (of phylum *Proteobacteria*) (Table S5). Thus, backbone protein families do not seem to follow host phylogeny. The emergence of dominant backbone proteins within a genus must therefore have been a more recent event.

Replication proteins, mobility proteins, and toxin antitoxin (TA) systems were the most dominant proteins, respectively. Finding replication and mobility proteins was expected, as they form the backbones of plasmids; however, finding several TA systems was not expected. TA systems can be thought of as selfish genetic elements that secure the stability of the plasmid and carry it through postsegregation killing (70). It could be speculated that the relative abundance of TA systems correlates to the small size distribution of our plasmids. The small sizes of the plasmids limit them to have barebones gene compositions. Replication genes, mobility genes, and TA systems allow the plasmids to be inherited, to spread, and to secure their persistence in the host, respectively.

### Global ARG occurrence.

The clustering of ARGs in the domain network correlated with their predicted hosts (Fig. S3) and their geographical origin (Fig. S4). RrnaAD and Aminoglyc_resit were found in Firmicutes members, and they lie in the mixed geographical cluster composed of rolling circle-replicating plasmids. ABC_tran, APH, and MFS_1 were found in the Acinetobacter plasmids and lie in the European cluster of theta-replicating plasmids (Fig. S3 and S4). The association between ARGs and genera makes sense, as not all ARGs are beneficial in all bacterial genera.

Pentapeptide_4 (and the member gene qnrB19) was not associated with any group in the domain network. Accordingly, it was associated with the Salmonella plasmids, which were not commonly predicted in the data. qnrB19-carrying plasmids seem to bear little resemblance to other plasmids, but they are found worldwide, especially in the Americas. The lack of plasmids similar to the qnrB19, together with the fact that the Salmonella-plasmids were not normally found in sewage, indicate that qnrB19 was not selected for in the wastewater environments.

The prevalence of macrolide resistance genes in sewage water across continents and species could indicate selective pressure for this trait in sewage water across the world. The genes msr(E) and mph(E) were almost exclusively located in the European samples. This could be a consequence of the composition of the microbiomes in these samples, as Acinetobacter, the carrier of the genes in sewage, was abundant in these samples. erm(B)-carrying plasmids were found worldwide, as were their host genera. It was recently indicated that methicillin resistance emerged in Staphylococcus aureus in hedgehogs as an adaptation to a β-lactam-producing dermatophyte (71). This highlights the coevolution among microbes in natural habitats as an important source of AMR emergence.

The prevalence of antibiotic resistance in wastewater has recently been reviewed (72). Uluseker et al. showed in their review that macrolide resistance is simply one of the ARGs that exists in wastewater in several previous studies. In a study comparing the abundances of ARGs via quantitative polymerase chain reaction (qPCR), ermA was not found to be notably more abundant than the other ARGs (73). This discrepancy could be a result of the selection bias toward short plasmids in our study. Overall, macrolide resistance may not be notably abundant in wastewater; instead, it may simply be the resistance type that is most likely to be carried on short plasmids. Another study by Che et al. reported that ARGs in the macrolide-lincosamide-streptogramin (MLS) group were more frequent on nonconjugative plasmids than on larger conjugative plasmids (74).

The emergence of AMR in wastewater has been studied collectively, and this has been done in Acinetobacter species, specifically. It was found that resistance to rifampin, chloramphenicol, and amoxicillin-clavulanic acid were significantly increased downriver of a wastewater treatment plant discharging point (7). However, the study did not include a macrolide in its screening panel, and the experiment only investigated a single treatment plant (in Ann Arbor, MI, USA). Others have shown a similar resistance increase at hospital, pharmaceutical plant, and wastewater treatment plant discharging points (73, 75–77). The general trend observed is that although wastewater treatment lowers bacterial counts, the prevalence of resistance among them increases.

A high percentage of antibiotics are not fully metabolized in humans, and macrolides have been detected in wastewater, although wastewater treatment plants lower their abundances (16, 78). The prevalence of various antibiotics in aquatic environments has recently been reviewed (79). Studies most commonly test for the presence of the trimethoprim-sulfamethoxazole combination used for urinary tract infections; however, the macrolide erythromycin was occasionally included in recent testing regimens. The mean concentration of the macrolide erythromycin in a large collection of aquatic environments was 1,507 ng/L (79). Two Welsh wastewater treatment plants, the only treatment plants included in the review, represented the highest detected concentrations, while drinking water represented the lowest concentration detected. However, the concentration of erythromycin was in the middle range compared to the other antibiotics reviewed in the study. Therefore, it does not explain the high prevalence of macrolide resistance found in this study.

Macrolide resistance selection apparently only affects Firmicutes and Acinetobacter species. This could be explained because of the limitations of machine learning models that predict bacterial genera, as the host bacterial genera in this article are based on predictions and are not experimentally verified. Nevertheless, it is notable that selective pressure toward macrolide resistance in sewage water seems to be a global phenomenon that affects different types of plasmids.

### Conclusion.

We found that the sewage water plasmidome was functionally distinct from sewage phages. There were a number of plasmid backbone gene combinations that were more common than others. These groups showed preferential cooccurrence with selected bacterial genera and signals of a geographical association with the sewage source (e.g., the larger numbers of bacteriophages and viruses compared to the number of plasmids in the Asian samples). Additionally, macrolide resistance-conferring plasmids were the most common in the plasmid data set. While msr(E) and mph(E) were mostly limited to Acinetobacter and the European samples, erm(B) appeared on all continents and throughout a number of genera, including important pathogens.

Macrolides are important antibiotics, and their emergence in the sewage water plasmidome could be a great threat to global health. The reason why macrolide resistance genes are the most prevalent has yet to be addressed, but it could be a consequence of the persistence of macrolides in wastewater compared to other compounds. Another factor is the species composition of the sewage water bacterial community. The common sewage water genus Acinetobacter carried a large proportion of the macrolide resistance genes. Therefore, the prevalence of this genus in sewage water to some extent explains the abundance of macrolide resistance in sewage plasmidomes. Though Acinetobacter is far from the most common human pathogen, the existence of a plasmid-carried reservoir of macrolide resistance is a cause for concern.

The worldwide occurrence of ARGs is an important aspect of global health management. This study shows how sewage plasmidomes can be surveyed for the emergence of ARGs in the environment, and it points to macrolide resistance as an important emergent gene in the global sewage plasmidome.

## MATERIALS AND METHODS

### Single read assembly.

Sewage water circular elements and their generation methods have been described in a previous publication (17). Briefly, sewage pellets were obtained by centrifuging the untreated sewage water from 24 samples from 22 countries, globally (full sample list is provided in Table S1). DNA was extracted from the sewage samples, the noncircular DNA was digested, and the circular DNA was amplified through rolling circle amplification. Libraries were prepared for both Oxford Nanopore and Illumina sequencing. Nanopore reads were trimmed, and short reads (<10 kb) were discarded. The raw reads consisted of tandem repeats from the rolling circle amplification step. These were assembled into the original circular sequence, which was then polished using Illumina reads with pilon (80).

### PLSDB comparison.

To compare our assemblies to known plasmids, we mapped them against the plasmid database PLSDB (version 2020_11_19 [81]) using nucleotide BLAST (blastn, all NCBI-blast commands were done with version 2.11.0+ [82]). Likewise, the PLSDB plasmids were BLASTed against the plasmid assemblies with NCBI-blast, this time, using the (PLSDB as the query and the assemblies as the subjects). If the PLSDB plasmid coverage and the assembly coverage were both above 90% for a given alignment, the pair was considered to be matching.

### Homology reduction.

Single read assembly creates duplicates, as each read from a plasmid can give rise to a sequence in the assembly. The assembly from each sample was homology reduced using kma (1.3.0) (83). The settings were: 80%. -k 16 -NI -Sparse - -ht 0.80 -hq 0.80 -and. A default k-mer size of 16 was used, and the homology thresholds were set to 80%. Homology reduction was done independently for each sample. The low thresholds were chosen as a countermeasure to the high error rate of the Oxford Nanopore sequencing.

### Phylogenetic analysis.

The longest 100 sequences from each of the 24 samples were individually sketched using MASH 2.2 (84), and a 2,400 by 2,400 distance table was calculated. Singletons (circular elements with a distance of 1 to all other circular elements) were removed from the table. A neighbor-joining tree was created using PHYLIP 3.697 (85) and visualized using Microreact (86).

### Pfam prediction and protein classification.

Circular elements were scanned for Pfam domains. These domain predictions have been used previously to classify elements as plasmids or phages (17). Briefly, Prodigal (version 2.6.3) (87) was used to predict proteins, which were then scanned against the Pfam database (version 33) (88) using hmmscan from HMMER (version 3.3.1) (89).

Pfam domains were used to classify the putative proteins on the circular elements (as in our previous publication, the E value cutoff was 0.000001) (17). If multiple domains with acceptable E values were predicted in a protein, the domain with the lowest E value was chosen. Hence, each protein was represented by its most significant domain. The number of times each domain was determined to be the most significant domain on a protein was counted. The proteins of the Comprehensive Antibiotic Resistance Database (CARD) (8) protein homolog model and a protein overexpression model had Pfam domains predicted in the same manner. The Pfam domains found on the CARD database proteins were added to our domain classification scheme as “Potential ARG”.

The combination of domains in each circular element was calculated. The amount of times a domain was found on each individual circular element was not considered. The amount of times each combination was encountered in a sample was counted.

We attempted to remove phage contamination with PPR-Meta (90) and SourceFinder (https://cge.cbs.dtu.dk/services/SourceFinder/.) (Fig. S1 and Fig. S2, respectively). However, neither method proved to be useful. PPR-Meta falsely predicted almost half of the plasmid sequences to be phage-derived, and SourceFinder predicted an equally large proportion of plasmids to be either chromosomal or phage-derived.

### Bipartite network graph.

A bipartite network of the circular elements and the domains found on them was constructed. For the circular elements, a node was made for each unique domain combination in each sample. For example, sixteen circular elements in the Indian sample only contained the domains Relaxase and MobC, and these were represented as a single node. For the domain part, each Pfam found in the data was simply represented as a node. An edge was drawn between a domain and a circular element if the domain was found on the circular element. To continue the example, MobC and Relaxase were each represented as a node, and the Indian node described above was connected to them both. The networks were visualized by Gephi (version 0.9.2) (91). The sizes of the nodes were estimated by their counts.

The colors of the nodes were based on Pfam function. This classification scheme was adapted from previous publications (17, 92). The scheme was updated to include toxin-antitoxin systems as a separate group and the potential ARG domains found in the CARD databases.

### ARG detection.

The macrolide resistance gene msr(E) contains an ABC transporter Pfam domain (ABC_tran). Thus, ABC_tran is marked as a potential ARG. However, not all ABC_tran-containing proteins are msr(E) or ARGs. To find bona fide ARGs, the homology-reduced circular elements were aligned against the ResFinder database (9) with kma (83), using the following parameters: bcNano on, bc = 0.7, mem_mode on.

### Plasmid host prediction.

The homology reduced circular elements were analyzed with a random forest model plasmid host predictor created by Aytan-Aktug et al. (51). The genus predicted with the highest probability was chosen as the host taxon.

### Data availability.

The data are available under ENA project accession number PRJEB41171 (finished assemblies are ERZ1694234 through ERZ1694257).

10.1128/msystems.00191-22.1 TEXT S1 Supplemental text file: Text on plasmid host prediction and DNA source prediction used in this study. Download Text S1, DOCX file, 0.02 MB. Copyright © 2022 Teudt et al. 2022 Teudt et al. https://creativecommons.org/licenses/by/4.0/ This content is distributed under the terms of the Creative Commons Attribution 4.0 International license.
