# Supplementary figures and images for: Global Distribution and Diversity of Prevalent Sewage Water Plasmidomes
Source: mSystems. 2022 Sep 7;7(5):e00191-22. doi: 10.1128/msystems.00191-22 (PMC9600348; doi:10.1128/msystems.00191-22)

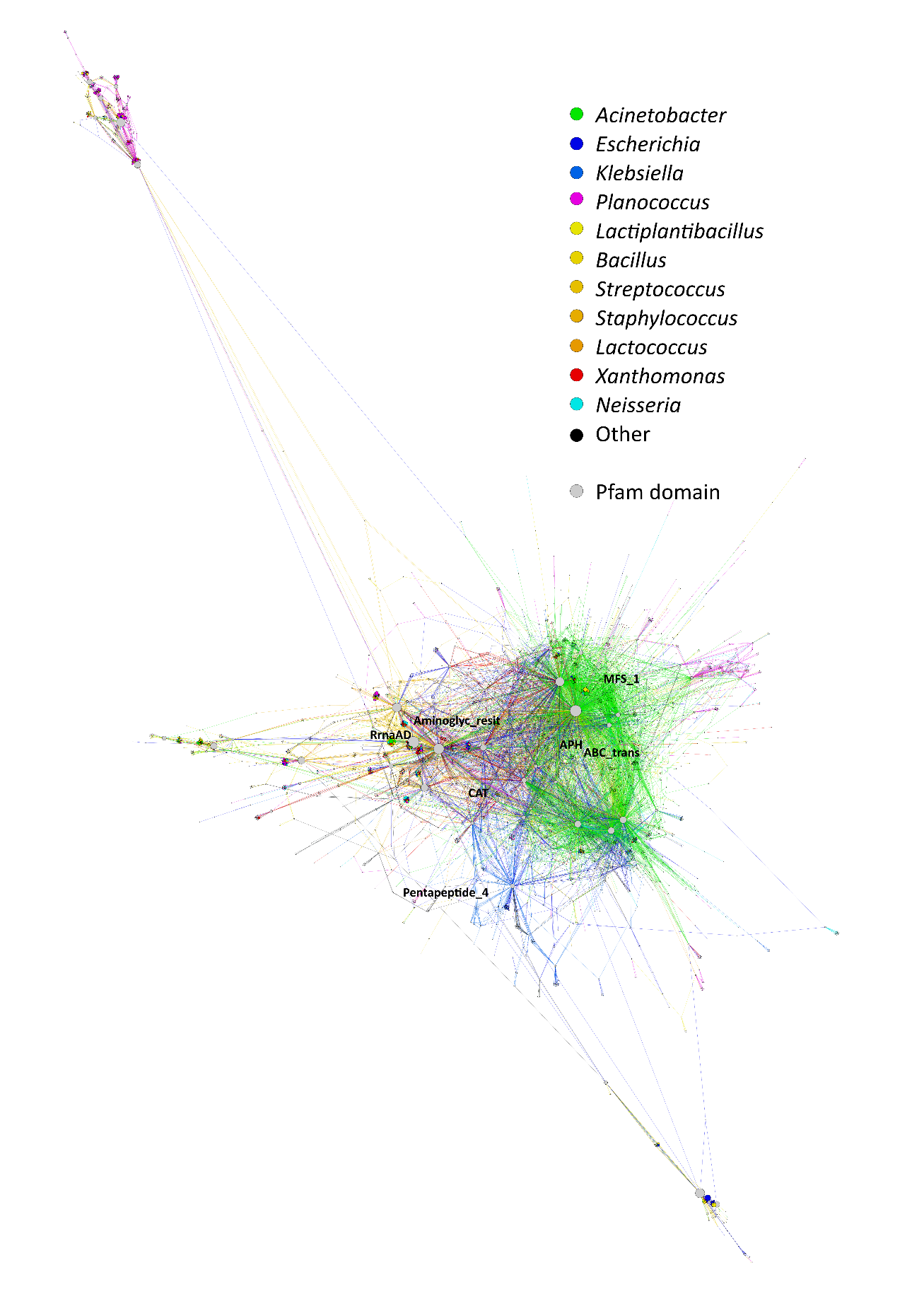

Supplement: FIG S3 [file msystems.00191-22-s0009.tif]

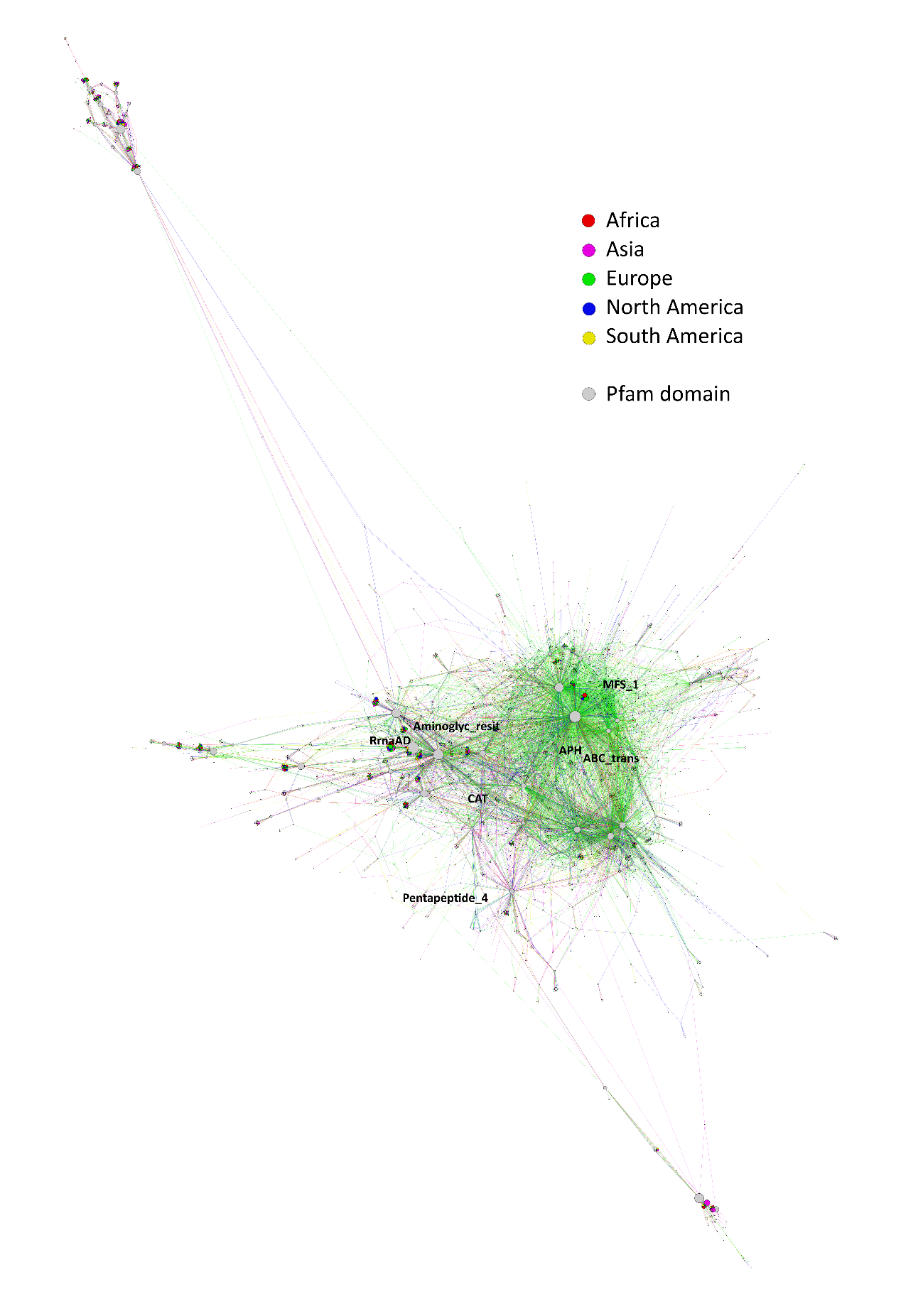

Supplement: FIG S4 [file msystems.00191-22-s0010.tif]
