# Supplementary material for: Global Distribution and Diversity of Prevalent Sewage Water Plasmidomes
Source: mSystems. 2022 Sep 7;7(5):e00191-22. doi: 10.1128/msystems.00191-22 (PMC9600348; doi:10.1128/msystems.00191-22)
Supplement: TEXT S1 [file msystems.00191-22-s0001.docx]

# Supplementary Information for

Global distribution and diversity of prevalent sewage water plasmidomes

Frederik Teudt^1^, Saria Otani^1^, Frank M. Aarestrup^1^

^1^Research Group for Genomic Epidemiology, Technical University of Denmark, Kgs. Lyngby, Denmark

Supplemental Table 1: List of the sewage samples that are included in the study. The data are available under ENA project accession number PRJEB41171 (finished assemblies are ERZ1694234 through ERZ1694257).

Supplemental Table 2: List of plasmid hosts predicted on sewage circular elements

Supplemental Table 3: Plasmid-encoded AMR genes and their predicted host organisms. AMR-containing plasmids had the genera of their hosts predicted. Counts indicate how many times a plasmid-encoded AMR gene was found in a given genus.

## Backbone gene tables for selected hosts

Supplemental Table 4: Backbone domains of five bacterial taxa: Planococcus (n=26165) backbone domains - Neisseria (n= 11710) backbone domains - Lactiplantibacillus (n= 10090) backbone domains - Acinetobacter (n= 8632) backbone domains - Escherichia (n= 5210) backbone domains.

## Plasmid host prediction

PlasmidHostFinder^1^ is a relatively new tool and we were unsure what parameters we should use. The predictor has a fragment model for metagenomic samples, where plasmids assemblies are expected to be incomplete. Though our samples are metagenomic, the plasmids are complete due to our unique assembly method, so we chose the non-fragment model. We validated this choice by cross-referencing with BLAST searches for a small plasmid subset (Supplemental Table 9 and Supplemental Table 10).

erm(B) and msr(E)-containing plasmids were BLASTed against the nt database using browser version of blastn from <https://blast.ncbi.nlm.nih.gov/Blast.cgi> (default parameters). The genus of the top hit was designated as the most likely host. The corresponding genus prediction from the non-fragment and fragment model for each plasmid was listed for comparison. It was clear the fragment model performed worst.

Supplemental Table 5: Host prediction of erm(B)-carrying plasmids. BLAST and the non-fragment model predicted all the plasmid hosts to be Firmicutes (with some disagreement about which genera specifically), while the fragment model predicted a far broader range of hosts, including non-Firmicutes. Followed by host prediction of msr(E)-carrying plasmids. BLAST and the non-fragment model predicted the plasmid hosts to be almost exclusively Acinetobacter.

## DNA source prediction

A subsample of sequences (the homology-reduced African samples) were chosen to evaluate potential ways to sort phages from plasmid. PPR-Meta^2^ and SourceFinder^3^ were tested. PPR-Meta was run with default settings, and SourceFinder was run with 10 sampling rounds. As ground truth, the Pfam domain predictions were used to identify sequences as originating from phages or plasmids (or NA if no signature domains were found on an element). Neither PPR-Meta predictions nor SourceFinder predictions corroborated with the ground truth for our dataset, so we decided against using these methods to filter the sequences.

Supplemental Figure 1: PPR-Meta predictions of DNA source for sequences with known phage domains (left facet), known plasmid domains (middle facet), and no signature domain (right facet).

Supplemental Figure 2: SourceFinder predictions of DNA source for sequences with known phage domains (left facet), known plasmid domains (middle facet), and no signature domain (right facet).

Distinguishing phages from plasmids were an issue. For circular elements with either plasmid domains or phage domains predicted on them, it was easy to determine the type. The lack of predicted backbone genes on a large proportion of the sequences meant we could not use backbone gene predictions for exhaustive classification of the circular elements, but we could use it as a control during the machine learning models. However, using predicted Pfam domains as a ground truth to compare with is not entirely correct. Rolling circle-replicating plasmids (replication group 1) were more closely associated with phages than the theta-replicating plasmids, presumably due to a slight overlap in domain use (Figure 4). However, this is the only apparent case where plasmid and phages are closely associated, thus it does not explain the high proportion of phages that PPR-Meta and SourceFinder predict in the subset of verified plasmids.

Supplemental Figure 3: The location of the most common ARG domains in the domain network with respect to predicted host genus.

Supplemental Figure 4: The location of the most common ARG domains in the domain network with respect to sample origin.

# Supplementary References

1. Aytan-Aktug, D. *et al.* PlasmidHostFinder: Prediction of plasmid hosts using random forest. *bioRxiv* 2021.09.27.462084 (2021). doi:10.1101/2021.09.27.462084

2. Fang, Z. *et al.* PPR-Meta: a tool for identifying phages and plasmids from metagenomic fragments using deep learning. *Gigascience* **8**, (2019).

3. https://cge.cbs.dtu.dk/services/SourceFinder/.
